# Multifaceted Determinants of Varicella Vaccination Uptake Among Children Aged 1–10 Years in China: Findings of a Population-Based Survey Among 996 Parents

**DOI:** 10.3390/vaccines13080810

**Published:** 2025-07-30

**Authors:** Weijun Peng, Yuan Fang, Hongbiao Chen, Minjie Zhang, Yadi Lin, Zixin Wang

**Affiliations:** 1 Longhua District Centre for Disease Control and Prevention, Shenzhen 518000, China; 2 Department of Health and Physical Education, The Education University of Hong Kong, Hong Kong, China; 3 Centre for Health Behaviours Research, JC School of Public Health and Primary Care, the Chinese University of Hong Kong, Hong Kong, China

**Keywords:** varicella vaccination, uptake, children, parents, parental stress, social media, family harmony, China

## Abstract

Background/Objectives: Chickenpox is an ongoing health threat for young children. This study aimed to investigate varicella vaccination uptake among children and its determinants at both the individual and interpersonal levels. Methods: A cross-sectional survey of parents of children aged 0–15 years and with administrative health records was conducted between September and October 2024 in Shenzhen, China. Participants were recruited through multistage random sampling. This analysis was based on a subsample of 996 parents whose children were 1–10 years old and without a prior history of chickenpox. Multivariate logistic regression models were fitted. Results: Among the participants, 47.0% reported that their children had received a varicella vaccination. Parents who believed that chickenpox was highly contagious (adjusted odds ratios [AOR]: 1.62, 95% confidence interval [CI]: 1.23, 2.13), perceived more benefits (AOR: 1.22, 95% CI: 1.05, 1.41) and cues to action (AOR: 1.33, 95% CI: 1.04, 1.69), and exhibited greater self-efficacy (AOR: 1.40, 95% CI: 1.09, 1.80) related to children’s varicella vaccination reported higher varicella vaccination uptake for their children. Greater perceived barriers related to vaccination (AOR: 0.89, 95% CI: 0.83, 0.95) and dysfunctional interactions with children (AOR: 0.97, 95% CI: 0.94, 0.99) were associated with lower varicella vaccination uptake for children. In addition, higher exposure to information encouraging parents to vaccinate their children against chickenpox (AOR: 1.24, 95%CI: 1.08, 1.41) and thoughtful consideration of the veracity of the information were associated with higher varicella vaccination uptake among children (AOR: 1.19, 95% CI: 1.05, 1.36). Conclusions: There is a strong need to promote varicella vaccination for children in China.

## 1. Introduction

Chickenpox is an acute and highly contagious infectious disease caused by the varicella-zoster virus and mainly affects children under the age of 12 years [1]. While the symptoms of chickenpox are usually mild, the infection could lead to severe complications, including pneumonia, encephalitis, and bacterial infections of the skin [1,2]. Although most people will develop lifelong immunity after natural infection, the virus may remain latent in the body and reactivate later, causing herpes zoster [2]. The World Health Organization (WHO) estimates that approximately 140 million children are affected by chickenpox every year, with 4.2 million of them having severe complications that require hospitalization and 4200 deaths [3]. In China, chickenpox is the third most frequently reported vaccine-preventable infectious disease and creates significant burdens on families and society [4]. The number of reported chickenpox cases in this country increased from 327,054 in 2010 to 981,699 in 2019 [4], and outbreaks of chickenpox are frequently reported in different Chinese cities, including in Shenzhen [5].

Receiving two doses of varicella vaccination can reduce the risk of chickenpox infection by 98% and is about 100% effective against severe complications of chickenpox [6]. The duration of protection is at least 10 years, without safety concerns [6]. Therefore, international health authorities recommend two doses of varicella vaccination for children, adolescents, and adults without evidence of immunity [6]. The WHO states that high coverage of varicella vaccination (i.e., 80%) should be achieved and maintained in countries where chickenpox poses a significant disease burden (e.g., China) [7]. Four types of varicella vaccines are available in mainland China. They are (1) Valrilrix (GlaxoSmithKline, GSK), (2) the varicella attenuated live vaccine manufactured by Shanghai Biological Products, (3) the varicella attenuated live vaccine from Changchun BCHT Biotechnology Co., and (4) V-SinoVac (SinoVac). With regard to varicella vaccines commonly used in western countries (e.g., Valrilrix [GSK] and Varivax [Merck Sharp & Dohme, MSD]), vaccines from manufacturers in mainland China have similar effectiveness in preventing clinical varicella [8,9]. Currently, varicella vaccination is not included in the national immunization program for children in mainland China, and receiving such vaccination is voluntary [10]. A two-dose schedule is implemented in this country: children can receive the first dose when they reach at least 12 months of age and the second dose at ≥4 years old [8]. Fully subsidized varicella vaccination has been implemented in Shenzhen, China for children aged 1–10 years since November 2019 [11].

There are large differences in varicella vaccination coverage among children across countries. Such coverage is usually inadequate in countries where varicella vaccination is not included in the national immunization program, such as 15% among Swedish children aged 1–8 years [12] or 8.2% among children aged 0–18 years in the United Kingdom [13]. After including varicella vaccination as a part of the national immunization plan, the coverage of such vaccines increased from 2.6–5.5% in 2006 to 86–100% among children aged 7 years in the United States [14]. In China, the overall coverage of varicella vaccination among children was 61.1% [15]. There is hence a need for improvement. Several studies have reported determinants of varicella vaccination uptake across countries, but not in mainland China. Facilitators of children’s varicella vaccination uptake included higher parental education and income, better parental knowledge about chickenpox, and more positive parental attitudes toward the vaccine (beliefs about vaccine efficacy and receiving recommendations from doctors and the government) [12,16,17,18]. Barriers to children’s varicella vaccination uptake included parental perceptions that chickenpox was a mild illness and natural immunity was superior and concerns about the safety and side effects of the vaccine [12,16,17,18]. These factors are considered in this study.

Parental stress is commonly experienced by parents when they deal with the roles of parenting. Previous studies have suggested that higher levels of parental stress are associated with lower quality of parenting [19]. In addition, parental stress can have significant and negative impacts on the health behaviors of both parents and children. Stressed parents may be more likely to engage in unhealthy behaviors as a way to cope with stress [20]. A systematic review suggested that mental health issues were associated with considerably lower vaccine uptake [21]. It is possible that parents with higher levels of parental stress are unlikely to vaccinate their children against infectious diseases. Chickenpox outbreaks, especially those occurring in school settings, are major topics on social media platforms in China [22]. Information exposure through different media channels may affect parents’ decisions to vaccinate their children against infectious diseases [23,24]. Previous studies have shown that higher exposure to experiences shared by vaccine recipients and information about the infectiousness of the virus was associated with higher parental acceptance of COVID-19 vaccination for their children [23]. Misinformation related to vaccination has been spreading rapidly on social media platforms, which is a known risk factor for vaccine hesitancy [25,26]. The extent of thoughtful consideration of the veracity of such information could mitigate the negative influences of such misinformation [27]. Previous studies have shown that a higher frequency of thoughtful consideration is associated with higher vaccination uptake [28]. Family harmony is a core value in traditional Chinese culture, which emphasizes peaceful, cooperative, and supportive family relationships. Previous studies have shown that better family harmony is associated with a lower occurrence of risky behaviors (e.g., smoking or drinking among youth) [29,30]. A more harmonious family may provide more social support to parents for child caring. Sufficient social support is a facilitator of vaccination uptake [31]. However, no study has tested the associations of parental stress, information exposure, or family harmony with children’s varicella vaccination uptake.

To address these knowledge gaps, this study investigated the influences of factors at the individual level (parental attitudes toward chickenpox and parental stress) and interpersonal level (parental exposure to information on social media platforms and family harmony) on children’s varicella vaccination uptake among parents in China. We hypothesized that variables at both the individual and interpersonal levels would be significantly associated with children’s varicella vaccination uptake.

## 2. Methods

### 2.1. Study Design

This was a secondary analysis of a cross-sectional survey among parents having at least one child aged 0–15 years, conducted during September to October 2024 in Shenzhen, China. Shenzhen, with a population of 17 million in 2021, is one of the four most developed cities (Beijing, Shanghai, Guangzhou, and Shenzhen) in mainland China. This analysis was based on a subsample of parents whose children were 1–10 years old and without a prior history of varicella infection. Fully subsidized varicella vaccination has been implemented in Shenzhen, China for children aged 1–10 years since November 2019.

### 2.2. Participants and Data Collection

The inclusion criteria of the original survey included (1) adults aged 18 years old or above, (2) having at least one child aged 0–15 years, and (3) having established administrative health records in community health centers. Since 2014, all Chinese residents who have lived in the same region for more than six months, regardless of age or permanent residency, have been required to establish administrative health records in local community health centers. The health records contain basic sociodemographic information, children’s development indicators, information related to pregnancy and childbirth (for women), chronic disease diagnoses and management, and vaccination history. It is expected that most adults who have lived in Shenzhen for more than six months have established administrative health records.

Multistage random sampling was used to recruit participants. The names of all 926 community health centers in Shenzhen were first entered into an Excel spreadsheet. Using the function of “selecting random cells”, ten community health centers were randomly selected. Within each selected community health center, 200 residents were randomly selected from the administrative health records of people who had at least one child aged 0–15 years. Staff in the selected health centers approached prospective participants through the telephone at different timeslots during weekdays and weekends, briefed them about the study, and invited them to visit the community health center to complete a face-to-face interview. Onsite, the staff of the community center obtained their written informed consent and performed the interview in Mandarin or Cantonese, which took about 30 min to complete. A cash coupon for CNY 25 (USD 3.5) was given to each participant after the completion of the interview. The current study obtained ethical approval from the Longhua District Centre for Disease Control and Prevention (CDC) (reference: 2024008).

A total of 996 participants were included in the secondary analysis. The uptake rate of varicella vaccination was approximately 50% among their children. Assuming the varicella vaccination uptake among their children in the reference group (without a facilitating condition) to be 10–40%, the sample size could detect the smallest odds ratios of 1.43 between parents with and without a facilitating condition, with power of 0.80 and an alpha of 0.05 (PASS 11.0, NCSS, LLC, Kaysville, the United States).

### 2.3. Measurements

#### 2.3.1. Development of the Questionnaire

A panel of experts in public health, vaccination behaviors, and health psychology, as well as CDC workers and parents, was formed to develop the questionnaire. The questionnaire was in simplified Chinese. We tested the readability and length of the questionnaire by conducting face-to-face interviews with 10 parents having at least one child aged 0–15 years. All participants in the pilot study reported that the questions were easy to understand, and the duration of the interview was acceptable. The panel finalized the questionnaire based on minor suggestions made by the participants.

#### 2.3.2. Background Characteristics

Participants reported their sociodemographic information (i.e., age group, sex assigned at birth, education level, employment status, history of SARS-CoV-2 infection, and seasonal influenza vaccination uptake) and the background characteristics of their children (i.e., age, sex assigned at birth, number of siblings, and seasonal influenza vaccination uptake). In the case of more than one child aged 0–15 years within the household, participants referred to the one whose birthday was closest to the survey date (the index child) when answering the questions.

#### 2.3.3. Outcome Variable: Index Child’s Varicella Vaccination Uptake

Participants were asked whether their index children had received any varicella vaccination. Some details related to varicella vaccination uptake (i.e., time and location) were collected from parents whose index children had received such vaccines.

#### 2.3.4. Independent Variables at the Individual Level

Three scales were constructed for this study to measure the perceived severity of chickenpox and the perceived benefits and barriers related to varicella vaccination. They were the 4-item Perceived Severity Scale (e.g., “Your index child will experience severe symptoms caused by chickenpox”), the 2-item Perceived Benefit Scale (e.g., “Varicella vaccination is effective in protecting your index child against chickenpox”), and the 3-item Perceived Barrier Scale (e.g., “Your index child will experience severe side effects after receiving a varicella vaccination”). The Cronbach’s alphas of these scales were between 0.77 and 0.82. In addition, four single items measured the perceived susceptibility (“Your index child has a high risk of having chickenpox”) and infectivity of chickenpox (“Chickenpox is highly contagious”) and perceived cues to action (“Your significant others will suggest you to have the index child received a varicella vaccination”) and self-efficacy related to varicella vaccination (“You are confident to have index child received a varicella vaccination”). The response categories for these items were 1 = disagree, 2 = neutral, and 3 = agree.

Parental stress was measured by the validated Chinese version of the Simplified Parenting Stress Index—Short Form (S-PSI-SF, 15 items) [32]. The S-PSI-SF consists of three subdomains, namely parenting distress, dysfunctional interaction, and difficult children. All items were rated on a 5-point Likert scale, from 1 being “strongly disagree” to 5 being “strongly agree”. Higher scores indicate higher levels of parental stress. The Cronbach’s alphas of the three subdomains ranged from 0.93 to 0.95.

#### 2.3.5. Independent Variables at the Interpersonal Level

Three items measured parents’ frequency of exposure to information related to chickenpox and varicella vaccination through different social media channels (e.g., WeChat, WeChat moments, Weibo, Red) in the past month. In addition, we modified a validated question to measure the frequency of the thoughtful consideration of the veracity of information related to varicella vaccination [27]. The aforementioned items were rated as 0 = almost none, 1 = seldom, 2 = sometimes, and 3 = often. Family harmony was measured by the validated Chinese version of the 5-item Family Harmony Scale [33]. All items were rated on a 5-point Likert scale, from 1 being “strongly disagree” to 5 being “strongly agree”. Higher scores indicate higher levels of family harmony. The Cronbach’s alpha of the Family Harmony Scale was 0.98 in this study.

### 2.4. Statistical Analysis

Descriptive statistics for all study variables are presented. Using varicella vaccination uptake among the index children as the dependent variable, and the background characteristics of the parents and index children as the independent variables, crude odds ratios (ORs) were obtained using logistic regression models. The associations between independent variables of interest (those at the individual and interpersonal levels) and the dependent variable were then investigated using a single logistic regression model involving one of the independent variables and all significant background characteristics. Adjusted odds ratios (AORs) and their corresponding 95% confidence intervals (CIs) were obtained. SPSS 26.0 (IBM Corp., Armonk, NY, USA) was used for the data analysis, and the significance level was set at *p* = 0.05 (2-sided).

## 3. Results

### 3.1. Background Characteristics of the Participants

Among the 2000 parents approached, 1504 of them completed the original survey (response rate: 75.2%), and 1352 of them had an index child aged 1–10 years. This analysis was based on 996 parents whose index child was 1–10 years old and without a prior history of varicella. Among these 996 parents, the majority of them were aged 31–40 years (65.3%), were the mothers of the index children (66.4%), had attained a tertiary education (80.3%), had full-time employment (68.4%), and had a monthly personal income of CNY 5000 (USD 697.4) or above (77.9%). About half of the index children were 4–6 years old (40.2%), male (51.7%), and had one sibling (56.4%). About 40% of the index children had received a seasonal influenza vaccination for the 2023/23 flu season (40.5%) (Table 1).

### 3.2. Varicella Vaccination Uptake and Descriptive Statistics of Independent Variables at the Individual and Interpersonal Levels

Among the parents, 47.0% reported that their index children had received a varicella vaccination. Regarding independent variables at the individual level, about half of the participants perceived that their index children had a high risk of chickenpox (45.1%). The majority of them perceived that their index children would have some severe consequences following chickenpox infection, such as severe symptoms among their children (52.8%) or affecting their children’s academic performance (73.6%) or their own work and income (71.6%). Most parents agreed that varicella vaccination was effective in protecting their index children (82.9%) and had a long duration of protection (68.8%), while about 30% of them were concerned about side effects and inconvenience related to the vaccine. Over three quarters of them perceived cues to action from significant others (75.9%) and were confident in having their index children receive varicella vaccination (78.4%). The means and standard deviations (SDs) of the scores of the three S-PSI-SF subdomains (i.e., parenting distress, dysfunctional interaction, and difficult children) are presented in Table 2.

Regarding independent variables at the interpersonal level, about half of the participants had sometimes or often been exposed to information encouraging parents to have their children vaccinated against varicella in the past month (49.6%), whereas fewer of them were exposed to information about chickenpox infection among classmates or peers of their index children (28.0%) or the situations or symptoms of children with chickenpox shared by their parents or caregivers (34.8%). Over half of the participants sometimes or often considered the veracity of information related to varicella vaccination (53.5%). The means and SDs of the scores of the Family Harmony Scale are shown in Table 2.

### 3.3. Factors Associated with Varicella Vaccination Uptake

In the univariate logistic regression analysis, parents who were older, female, permanent Shenzhen residents, and reported seasonal influenza vaccination uptake for the 2023/24 flu season were more likely to report varicella vaccination uptake for their index children. In addition, index children who were older, female, had one sibling, and received a seasonal influenza vaccination for the 2023/24 flu season had a higher uptake rate of varicella vaccination (Table 3).

After adjusting for these significant background characteristics, parents who believed that chickenpox was highly contagious (AOR: 1.62, 95% CI: 1.23, 2.13, *p* < 0.001), perceived more benefits of varicella vaccination for the index children (AOR: 1.22, 95% CI: 1.05, 1.41, *p* = 0.01), received more cues to action (AOR: 1.33, 95% CI: 1.04, 1.69, *p* = 0.02), and had higher self-efficacy related to the index children’s varicella vaccination (AOR: 1.40, 95% CI: 1.09, 1.80, *p* = 0.01) were more likely to report varicella vaccination uptake for their index children. Greater perceived barriers related to index children’s varicella vaccination (AOR: 0.89, 95% CI: 0.83, 0.95, *p* < 0.001) and dysfunctional interactions with the children (AOR: 0.97, 95% CI: 0.94, 0.99, *p* = 0.01) were associated with lower varicella vaccination uptake for the index children. Regarding interpersonal-level variables, higher exposure to information encouraging parents to have their children vaccinated against varicella (AOR: 1.24, 95% CI: 1.08, 1.41, *p* = 0.002) and a higher frequency of thoughtful consideration of the veracity of the information (AOR: 1.19, 95% CI: 1.05, 1.36, *p* = 0.01) were associated with higher varicella vaccination uptake among the index children (Table 4).

## 4. Discussion

To our knowledge, this is one of the first studies to investigate the influences of parental stress, information exposure through social media platforms, and family harmony on children’s varicella vaccination uptake. The findings extend the application of socioecological models and provide practical implications to inform health promotion. The results also represent the latest estimates of varicella vaccination coverage among children who are eligible to receive fully subsidized varicella vaccination in Shenzhen, China. Such data on vaccination coverage are useful to evaluate the effectiveness of varicella vaccination programs implemented by the government. Other strengths of this study include the relatively large sample recruited by multistage random sampling.

In this study, 47% of children aged 1–10 years received at least one dose of varicella vaccination. Such coverage was slightly lower than the overall varicella vaccination coverage among children in mainland China [15] and was much lower than in regions or countries where varicella vaccination is a part of the national immunization plan for children (e.g., United States) [14]. More efforts are needed to achieve the WHO-recommended varicella vaccination coverage of 80% [7]. Given the heavy disease burdens caused by chickenpox, health authorities in mainland China should consider including varicella vaccination in the national immunization program for children.

Older children had higher varicella vaccination uptake. Such a trend was expected as the study outcome was the accumulated number of children vaccinated at the time of the survey. Girls had higher varicella vaccination uptake than boys. Health communication messages should emphasize that boys and girls have similar risks of developing severe complications following chickenpox infection [34]. Parents who were permanent residents of Shenzhen reported higher varicella vaccination uptake for their children. Access to health services is one of the challenges faced by internal migrants upon arrival in a new city, due to unfamiliarity with the local system and health insurance complexities [35]. Therefore, focused attention should be given to parents who are internal migrants in future health promotion. Having a sibling was associated with higher vaccination uptake among children. Parents might be concerned about the risk of chickenpox transmission between siblings and hence are more motivated to vaccinate their children against this disease. Seasonal influenza vaccination uptake among both parents and children was associated with higher varicella vaccination uptake among children. Previous studies have found strong associations between parental and children’s seasonal influenza and HPV vaccination uptake [36,37]. Receiving seasonal influenza vaccination is voluntary and self-financed for adults under the age of 60 years in mainland China. Parents with a recent history of seasonal influenza vaccination might have more positive attitudes toward vaccination, leading them to immunize their children against different infectious diseases [34]. Moreover, parental vaccination behaviors might motivate children to follow their examples.

Our study provides some practical implications to develop interventions promoting varicella vaccination for children. At the individual level, health promotion may consider modifying parental attitudes toward chickenpox and varicella vaccination. Among the parents, 78.8% perceived that chickenpox was highly contagious, 82.9% perceived that varicella vaccination was effective in protecting children against chickenpox, and 68.8% agreed that the duration of protection offered by the vaccine was long. It would be useful to strengthen these perceptions, as they are associated with higher varicella vaccination uptake among children, and there is still room for improvement. Over 30% of the parents perceived some barriers to having their children receive a varicella vaccination, and perceived barriers were associated with lower varicella vaccination uptake among their children. To address these barriers, health communication messages should emphasize that the common adverse effects of varicella vaccination are mild. Testimonials shared by parents of vaccinated children regarding children’s experiences of varicella vaccination may be helpful, as parents often value the experiences and perspectives of other parents [38]. Moreover, health communication messages should highlight that, although people can develop lifelong immunity after natural infection with chickenpox, the virus may remain latent in the body and be reactivated later, causing herpes zoster [2]. In addition, information on the locations and working hours of facilities providing fully subsidized varicella vaccination to children should be disseminated to parents to eliminate concerns about inconvenience. Future programs should also involve the significant others of parents, such as doctors, other family members, and peers, as receiving cues to action from these people is associated with higher varicella vaccination among their children. Providing outreach vaccination services in kindergartens and primary schools and facilitating action planning may be useful strategies to improve perceived self-efficacy among parents, another variable that is associated with higher varicella vaccination uptake among their children. Dysfunctional interaction, a subdomain of parental stress measuring parents’ perceptions that their children do not meet their expectations and that their interactions with the children are not reinforcing to them as parents, was associated with lower varicella vaccination among children [32]. Helping parents to develop positive parenting strategies, including effective communication with children, may be helpful to reduce parental stress due to dysfunctional interactions [39].

At the interpersonal level, parents who had a higher frequency of exposure to information encouraging parents to have their children vaccinated against varicella were more likely to report varicella vaccination for their children. Such findings are similar to those of previous studies on parental acceptance of COVID-19 and seasonal influenza vaccination [23,24]. Previous studies have suggested that information exposure through different media channels can affect attitudes toward health behaviors and hence affect the occurrence of such behaviors [40,41]. Therefore, future programs may consider using popular social media platforms, which have good reach among Chinese parents, to disseminate health communication messages promoting varicella vaccination. In line with previous studies, parents who more frequently considered the veracity of information were more likely to vaccinate their children against chickenpox [28]. It is necessary to enhance the parents’ capacity and resources to analyze and appraise the obtained information. There is a need for intervention, as only half of the parents in this study always considered the veracity of information related to varicella vaccination.

This study had several limitations. First, we did not collect information from parents who refused to join the study. It is possible that participants and those who refused to participate had different characteristics. Self-selection bias existed. Second, the history of varicella vaccination is not available in the administrative health records as such vaccination is not a part of the national immunization plan. Therefore, we were not able to validate children’s varicella vaccination uptake as reported by parents. Since the interviews were conducted in community health centers, taking up a vaccine might have been considered socially desirable by the respondents [42]. Participants might have overreported varicella vaccination uptake among their children due to social desirability bias. Third, the items and scales measuring perceptions related to chickenpox and varicella vaccination were self-constructed due to a lack of validated measurements in the Chinese context. The Cronbach’s alphas of these scales were acceptable. However, these items or scales were not validated in a separate study. Fourth, due to differences in healthcare systems, vaccination delivery models, health literacy, and cultural factors, the findings might not be applicable to other cities in China. Shenzhen is one of the most developed cities in mainland China. It is expected that the performance of its healthcare system and its residents’ health literacy would be higher than those in less developed regions of China. Therefore, more parents in Shenzhen might be willing to vaccinate their children against chickenpox compared to their counterparts living in less developed regions. Moreover, causality could not be established as this was a cross-sectional survey.

## 5. Conclusions

Despite the free vaccine, the coverage of varicella vaccination remains inadequate among Chinese children. Future health programs may consider modifying parents’ attitudes related to chickenpox and varicella vaccination and helping parents to develop positive parenting strategies to reduce parental stress related to dysfunctional interactions. Disseminating health communication messages through social media platforms may also be useful.

## Figures and Tables

**Table 1:** Background characteristics of the participants and their index children.

	n	%
**Background characteristics of parents**		
Parent’s age group, years		
24–30	117	11.7
31–40	650	65.3
41–55	229	23.3
Parent’s sex assigned at birth		
Male	335	33.6
Female	661	66.4
Parent’s education level		
Secondary or below	196	19.7
Tertiary or above	800	80.3
Parent’s current relationship status		
Married	967	97.1
Single/divorced/separated/widowed	29	2.9
Parent’s current employment status		
Full-time	581	68.4
Part-time/self-employed/unemployed/housewife	315	31.6
Parent’s monthly personal income level		
Below CNY 5000 (USD)	220	22.1
CNY 5000–9999 (USD)	361	36.2
CNY 10,000 (USD) or above	415	41.7
Parent’s history of confirmed SARS-CoV-2 infection		
No	191	19.2
Yes	805	80.2
Parent’s seasonal influenza vaccination uptake for the 2023/24 flu season		
No	831	83.4
Yes	165	16.6
**Background characteristics of the index child**		
Index child’s age group, years		
1–3	244	24.5
4–6	400	40.2
7–10	352	35.3
Index child’s sex assigned at birth		
Male	515	51.7
Female	481	48.3
Number of siblings		
0	354	35.5
1	562	56.4
2	80	8.0
Index child’s seasonal influenza vaccination uptake for the 2023/24 flu season		
No	593	59.5
Yes	403	40.5

**Table 2:** Varicella vaccination uptake and descriptive statistics of independent variables of interest.

	n	%
**Varicella vaccination uptake**		
Index child has taken up any varicella vaccination		
No	528	53.0
Yes	463	47.0
**Attitudes toward varicella vaccination**		
Perceived susceptibility to chickenpox, agree		
Your index child has a high risk of having chickenpox	449	45.1
Item score, mean (SD)	2.2	0.8
Perceived severity of chickenpox, agree		
Your index child will experience severe symptoms caused by chickenpox	526	52.8
The chickenpox virus can remain in the body for a long time and recur many years later, causing severe symptoms	554	55.6
If your index child gets chickenpox, it will seriously affect his/her academic performance	733	73.6
If your index child gets chickenpox, you will have to take care of him/her and your work and income will be affected	713	71.6
Perceived Severity Scale ^1^		
Scale score, mean (SD)	10.1	2.2
Perceived benefit of varicella vaccination, agree		
Varicella vaccination is effective in protecting your index child against chickenpox	826	82.9
The duration of protection of varicella vaccination is very long	685	68.8
Perceived Benefit Scale ^2^		
Scale score, mean (SD)	5.4	1.0
Perceived barriers to receive a varicella vaccination, agree		
Your index child will experience severe side effects after receiving a varicella vaccination	374	37.6
Your index child will not get chickenpox twice, there is no need to receive a vaccine	383	38.5
You do not know where to take the index child to receive a varicella vaccination	332	33.3
Perceived Barrier Scale ^3^		
Scale score, mean (SD)	6.2	2.0
Perceived cue to action to receive a varicella vaccination, agree		
Your significant others (e.g., doctors, family members or friends) will suggest that you have the index child receive a varicella vaccination	756	75.9
Item score, mean (SD)	2.7	0.6
Perceived self-efficacy to receive a varicella vaccination, agree	781	78.4
You are confident to have the index child receive a varicella vaccination		
Item score, mean (SD)	2.7	0.6
Perceived infectivity of chickenpox, agree		
Chickenpox is highly contagious	785	78.8
Item score, mean (SD)	2.7	0.5
**Parenting stress**		
Parenting distress subdomain of the Simplified Parenting Stress Index—Short Form ^4^		
Scale score, mean (SD)	15.3	5.1
Dysfunctional interaction subdomain of the Simplified Parenting Stress Index—Short Form ^5^		
Scale score, mean (SD)	12.3	5.6
Difficult child subdomain of the Simplified Parenting Stress Index—Short Form ^6^		
Scale score, mean (SD)	12.7	5.4
**Information exposure related to chickenpox**		
Frequency of exposure to the following information through different social media channels (e.g., WeChat, WeChat moments, Weibo, TikTok, Red, etc.)		
Many classmates/peers of the index child have chickenpox		
Almost none	326	32.7
Seldom	391	39.3
Sometimes	217	21.8
Often	62	6.2
Item score, mean (SD)	1.0	0.9
After children got chickenpox, the parents/caregivers shared their situations or symptoms		
Almost none	330	33.1
Seldom	319	32.0
Sometimes	256	25.7
Often	91	9.1
Item score, mean (SD)	1.1	1.0
Information encouraging parents to have their children receive varicella vaccination		
Almost none	225	22.6
Seldom	277	27.8
Sometimes	291	29.2
Often	203	20.4
Item score, mean (SD)	1.5	1.1
Thoughtful consideration of the veracity of information related to varicella vaccination		
Almost none	217	21.8
Seldom	246	24.7
Sometimes	319	32.0
Often	214	21.5
Item score, mean (SD)	1.5	1.1
**Family harmony**		
Family Harmony Scale ^7^		
Scale score, mean (SD)	19.8	4.3

^1^ Perceived Severity Scale, 4 items, Cronbach’s alpha: 0.82. ^2^ Perceived Benefit Scale, 2 items, Cronbach’s alpha: 0.79. ^3^ Perceived Barrier Scale, 3 items, Cronbach’s alpha: 0.77. ^4^ Parenting distress subdomain of the Simplified Parenting Stress Index—Short Form, 5 items, Cronbach’s alpha: 0.93. ^5^ Dysfunctional interaction subdomain of the Simplified Parenting Stress Index—Short Form, 5 items, Cronbach’s alpha: 0.95. ^6^ Difficult child subdomain of the Simplified Parenting Stress Index—Short Form, 5 items, Cronbach’s alpha: 0.95. ^7^ Family Harmony Scale, 5 items, Cronbach’s alpha: 0.98.

**Table 3:** Associations between background characteristics and index child’s uptake of varicella vaccination.

	OR (95%CI)	*p* Values
**Background characteristics of parents**		
Parent’s age group, years		
24–30	Reference	
31–40	1.69 (1.12, 2.55)	0.01
41–55	1.94 (1.22, 3.07)	0.01
Parent’s sex assigned at birth		
Male	Reference	
Female	1.62 (1.24, 2.12)	<0.001
Parent’s education level		
Secondary or below	Reference	
Tertiary or above	1.11 (0.81, 1.52)	0.51
Parent’s current relationship status		
Married	Reference	
Single/divorced/separated/widowed	1.22 (0.58. 2.55)	0.61
Parent’s current employment status		
Full-time	Reference	
Part-time/self-employed/unemployed/housewives	0.98 (0.75, 1.28)	0.89
Parent’s monthly personal income level		
Below CNY 5000 (USD)	Reference	
CNY 5000–9999 (USD)	0.87 (0.62, 1.21)	0.40
CNY 10,000 (USD) or above	1.01 (0.73, 1.40)	0.95
Whether parent is a permanent Shenzhen resident		
No	Reference	
Yes	1.62 (1.24, 2.12)	<0.001
Parent’s history of confirmed SARS-CoV-2 infection		
No	Reference	
Yes	1.26 (0.91, 1.73)	0.16
Parent’s seasonal influenza vaccination uptake for the 2023/24 flu season		
No	Reference	
Yes	3.01 (2.11, 4.31)	<0.001
**Background characteristics of the index child**		
Index child’s age group, years		
1–3	Reference	
4–6	1.55 (1.12, 2.15)	0.01
7–10	1.53 (1.10, 2.14)	0.01
Index child’s sex assigned at birth		
Male	Reference	
Female	1.30 (1.01, 1.66)	0.04
Number of siblings		
0	Reference	
1	1.52 (1.16, 1.98)	0.002
2	1.12 (0.69, 1.83)	0.65
Index child’s seasonal influenza vaccination uptake for the 2023/24 flu season		
No	Reference	
Yes	4.37 (3.33, 5.73)	<0.001

OR: crude odds ratios. CI: confidence interval.

**Table 4:** Factors associated with index child’s uptake of varicella vaccination.

	AOR (95%CI)	*p* Values
**Attitudes toward varicella vaccination**		
Your index child has a high risk of having chickenpox (perceived susceptibility)	0.90 (0.76, 1.07)	0.25
Perceived Severity Scale	1.00 (0.94, 1.06)	0.90
Perceived Benefit Scale	1.22 (1.05, 1.41)	0.01
Perceived Barrier Scale	0.89 (0.83, 0.95)	<0.001
Your significant others (e.g., doctors, family members or friends) suggest that you to have the index child receive a varicella vaccination (perceived cue to action)	1.33 (1.04, 1.69)	0.02
You are confident in having your index child receive a varicella vaccination (perceived self-efficacy)	1.40 (1.09, 1.80)	0.01
Chickenpox is highly contagious	1.62 (1.23, 2.13)	<0.001
**Parenting stress**		
Parenting distress subdomain of the Simplified Parenting Stress Index—Short Form	1.00 (0.97, 1.02)	0.76
Dysfunctional interaction subdomain of the Simplified Parenting Stress Index—Short Form	0.97 (0.94, 0.99)	0.01
Difficult child subdomain of the Simplified Parenting Stress Index—Short Form	0.98 (0.95, 1.00)	0.07
**Information exposure related to chickenpox**		
Frequency of exposure to the following information through different social media channels (e.g., WeChat, WeChat moments, Weibo, TikTok, Red, etc.)		
Many classmates/peers of the index child have chickenpox	0.94 (0.80, 1.09)	0.41
After children got chickenpox, the parents/caregivers shared their situations or symptoms	0.97 (0.84, 1.11)	0.63
Information encouraging parents to have their children receive varicella vaccination	1.24 (1.08, 1.41)	0.002
Thoughtful consideration of the veracity of information related to varicella vaccination	1.19 (1.05, 1.36)	0.01
**Family harmony**		
Family Harmony Scale	1.02 (0.99, 1.05)	0.23

AOR: adjusted odds ratios—odds ratios adjusted for significant background characteristics listed in Table 3.

## Data Availability

The datasets generated and/or analyzed during the current study are not publicly available as they contain sensitive personal information, but they are available from the corresponding author upon reasonable request.
